# Two closely spaced microstrip patches with high isolation for full-duplex/MIMO applications

**DOI:** 10.1371/journal.pone.0290980

**Published:** 2023-10-09

**Authors:** Huy-Hung Tran, Tung The-Lam Nguyen, Thuy Nguyen Thi

**Affiliations:** 1 Faculty of Electrical and Electronic Engineering, PHENIKAA University, Hanoi, Vietnam; 2 IT Department, FPT University, Greenwich Vietnam, Hanoi, Vietnam; 3 RnD Center, ESSYS Co., Ltd, Incheon, Korea; Edinburgh Napier University, UNITED KINGDOM

## Abstract

This paper shows an effective method to significantly enhance the isolation of a closely spaced two-port patch antenna, which can be deployed for full-duplex transceivers as well as multiple-input-multiple-output (MIMO) systems. Two rectangular microstrip patches are arranged in the E-plane configuration. To achieve high isolation, a grounded stub is positioned between the radiating patches. For validation of the proposed concept, an antenna prototype is fabricated for measurements. The measured data demonstrates that the port-to-port mutual coupling can be suppressed to –50 dB, which is useful in self-interference cancellation for full-duplex communication systems. Compared with the coupled design, the isolation is significantly enhanced by 43 dB with an inter-element spacing of 0.034λ_*c*_, where λ_*c*_ is a free-space wavelength at the center operating frequency. Regarding MIMO metrics, the antenna also shows good MIMO diversity performance based on an envelope correlation coefficient and a diversity gain.

## Introduction

Full-duplex antenna systems can provide high data rate wireless communication. This kind of technology utilizes the same time/frequency slot for both reception and transmission, which help to enhance spectral efficiency in comparison with the half-duplex communication systems [1]. To reduce the cost, an effective method is to use the passive antenna with mutual coupling reduction in the full-duplex antenna systems. Besides, a multiple-input multiple-output (MIMO) antenna is another enabling technology for advanced 4G/5G wireless communication [2]. As modern electronic devices are getting smaller, the antenna is arranged in a constrained space, leading to high mutual coupling between the radiating elements. This paper focuses on designing a two-port antenna with high isolation and small inter-element spacing, which can be deployed for both full-duplex and MIMO systems.

Among many types of antenna structures, a microstrip patch antenna is widely used due to its low-profile, low cost, and ease of integration characteristics [3, 4]. Various techniques for coupling reduction in multiple-element antenna arrays have been reported in the literature [5]. The principle for high isolation is to employ a decoupling network to suppress the surface-wave and space-wave coupling, which are occupied inside and outside the substrate. It is noted that the position of the decoupling network is a critical factor in determining the compactness of the antenna system.

The first decoupled type is to locate the network in a different substrate with the radiating elements. For example, the authors proposed methods of using near-field resonators (NFRs) [6, 7] and metasurfaces (MSs) [8, 9], which are placed above the radiating elements. Although high isolation of 40 dB can be achieved by controlling the space-wave coupling, the NFRs, and MSs significantly increase the antenna profile to higher than 0.15λ_*c*_ (λ_*c*_ is a free-space wavelength at the center operating frequency). Alternatively, although using a dielectric block in [10] to cancel the surface-wave coupling is another effective method, it’s only suitable for antennas operating at very high-frequency bands due to the use of a thick substrate. The decoupling network could be positioned underneath the radiating elements [11] or in the vertical direction [12, 13]. The scheme in [11] makes a mathematical analysis of the scattering parameters (S-parameters). In [12, 13], a post-wall slot line and a coplanar strip wall act as a band-stop filter. Nonetheless, high isolation is attained at the cost of increasing the complexity of the feeding circuit or the antenna profile, which is up to 0.22λ_*c*_.

The second type is to put the decoupling structures on the same substrate with the radiating elements. For example, defected ground structures (DGSs) working as band-stop filters are inserted into the ground plane [14, 15]. The designs with DGS can achieve small edge-to-edge spacing of 0.03λ_*c*_. However, the DGS causes high back radiation due to ground disturbance. Another band-stop structure employs metamaterial (MTMs) [16–19], which are positioned in the same layer with the radiating patches. Nonetheless, large element spacing of about 0.2λ_*c*_ is required. In [20, 21], a technique of utilizing neutralization lines (NLs) to create an extra coupling path to cancel the original coupling is proposed. In [22], a lump inductance is employed for coupling reduction. The advantage of MTMs and NLs is that the element spacing can be reduced to less than 0.02λ_*c*_. However, high cross-polarization is a critical drawback of the design [18] and low isolation is the main disadvantage of the antenna reported in [22]. Alternatively, embedding linear slots near the periphery of the patch [23] or rectangular loops [24], or a comb-shaped structure [25, 26] are also demonstrated as other effective solutions with large element spacing.

In this paper, a closely packed two-port microstrip patch antenna with high isolation and small element spacing is presented. The proposed decoupling network is a single stub, which is grounded through two shorting vias. This method has been demonstrated as an effective technique to significantly reduce the mutual coupling of the two-element E-plane antenna. The fabricated design with an edge-to-edge spacing of 0.034λ_*c*_ can improve the isolation from 7 to 50 dB. Compared to the other reported designs, the proposed antenna has the advantage of high isolation while possessing very small element spacing.

## Geometry of two-element MIMO antenna

**Fig 1:**
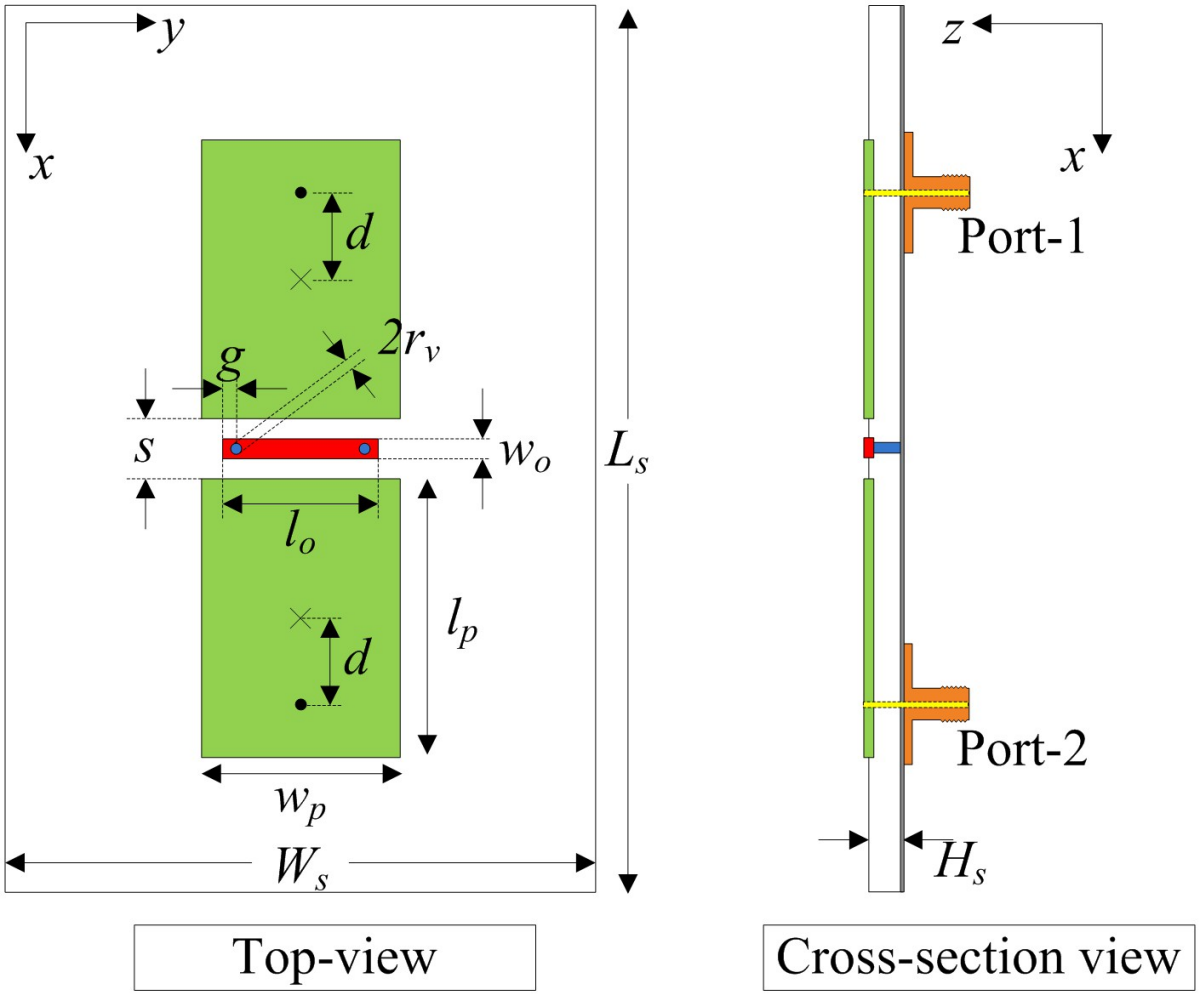
Geometry of the proposed two-port patch antenna.

The geometry of a two-element array with a decoupling network is presented in Fig 1. The microstrip patches are printed on the top side of a TLY-5 Taconic substrate with a relative permittivity of 2.2 and a loss tangent of 0.0009. The patches are arranged in the E-plane configuration with an edge-to-edge distance of 2 mm, corresponding to 0.034 λ_*c*_ at 5.2 GHz. For coupling reduction, a single stub is placed between the two patches, and two vias are utilized to connect the stub to the ground plane. The length of the patch (*l*_*p*_) is defined as Eq (1):

$$\begin{array}{c} l_{p}=\frac{c}{2f_{r}\sqrt{\varepsilon_{eff}}} \end{array}$$ (1)

where *f*_*r*_ is the desired operating frequency and *ε*_*eff*_ is the effective permittivity of the used substrate.

The antenna is simulated and characterized in the full-wave High-Frequency Structure Simulator (HFSS) and then validated by measurement. The antenna is optimized for the best performance in terms of operating bandwidth (BW) and inter-port isolation. The overall dimensions are 60 mm × 40 mm for all cases. The optimal dimensions of the proposed design are given in Table 1 as Antenna-4.

**Table 1:** Optimized dimensions of different antenna configurations (unit: mm).

Parameter	Antenna-1	Antenna-2	Antenna-3	Antenna-4
*l*_*p*_	18	16.8	16.8	16.8
*w*_*p*_	14.8	14.8	14.8	14.8
*d*	3.6	3.2	3.2	3.2
*s*	2	2	2	2
*l*_0_		4.8	4.1	7.9
*w*_0_		1	1	1
*g*		0.5	0.5	0.5
*r*_*v*_		0.25	0.4	0.2

## Antenna design

### Antenna design process

**Fig 2:**
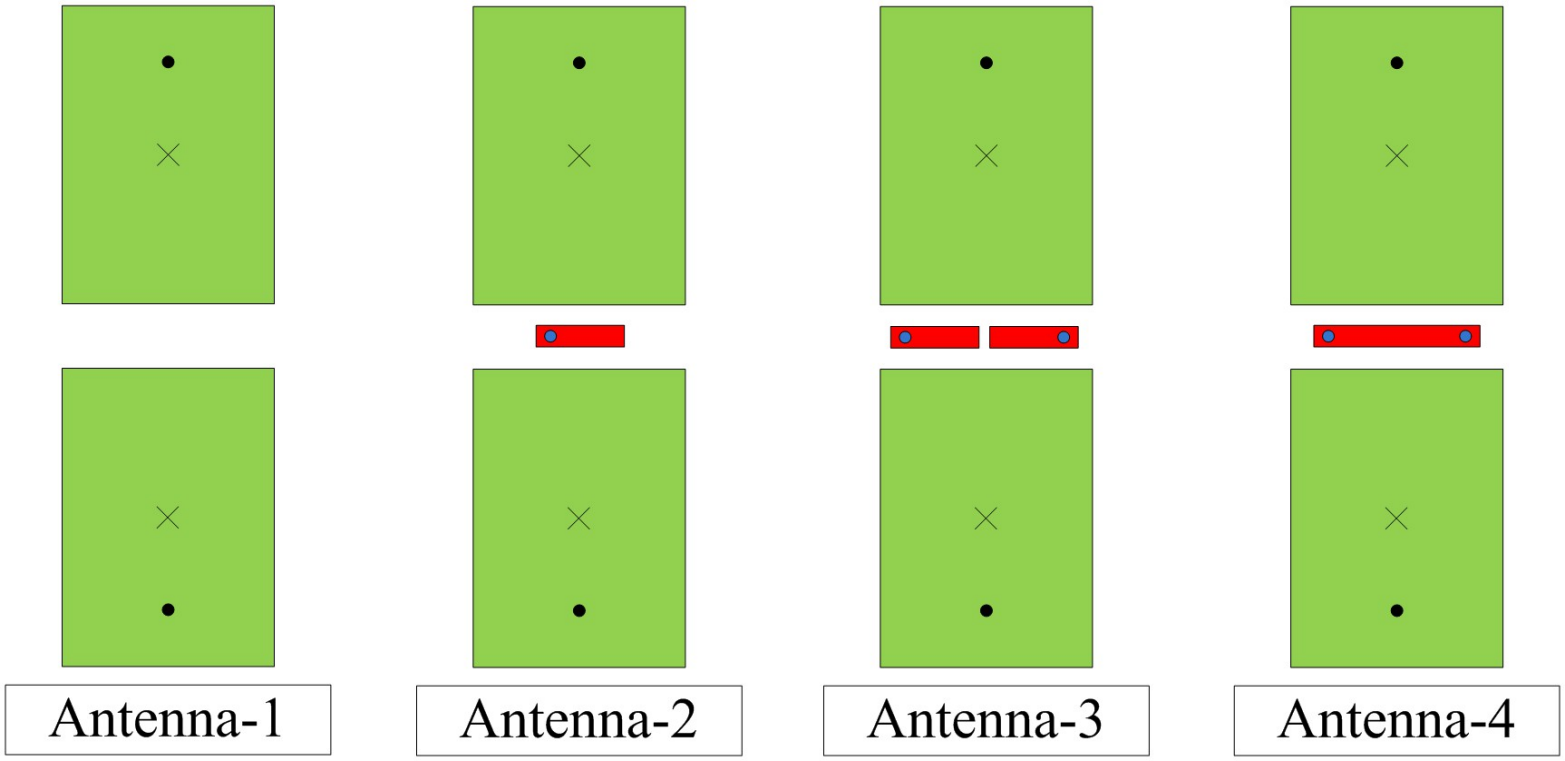
Design process of the proposed two-port patch antenna.

To demonstrate the effectiveness of the proposed decoupling method, a performance comparison between different antennas with and without a decoupling network is considered. Note that the coupled design is optimized to operate at a similar band to the decoupled designs. Fig 2 shows the steps to achieve the final realization of the proposed two-port antenna. First, the coupled antenna without a decoupling network is denoted as Antenna-1. Next, the antenna with a decoupling network formed by a single stub and a single via is designated as Antenna-2. For Antenna-3, one more grounded stub is utilized for better coupling suppression. Finally, Antenna-4 with connected grounded stubs is realized. These designs are optimized for operation in a similar frequency range. The optimized dimensions are presented in Table 1.

**Fig 3:**
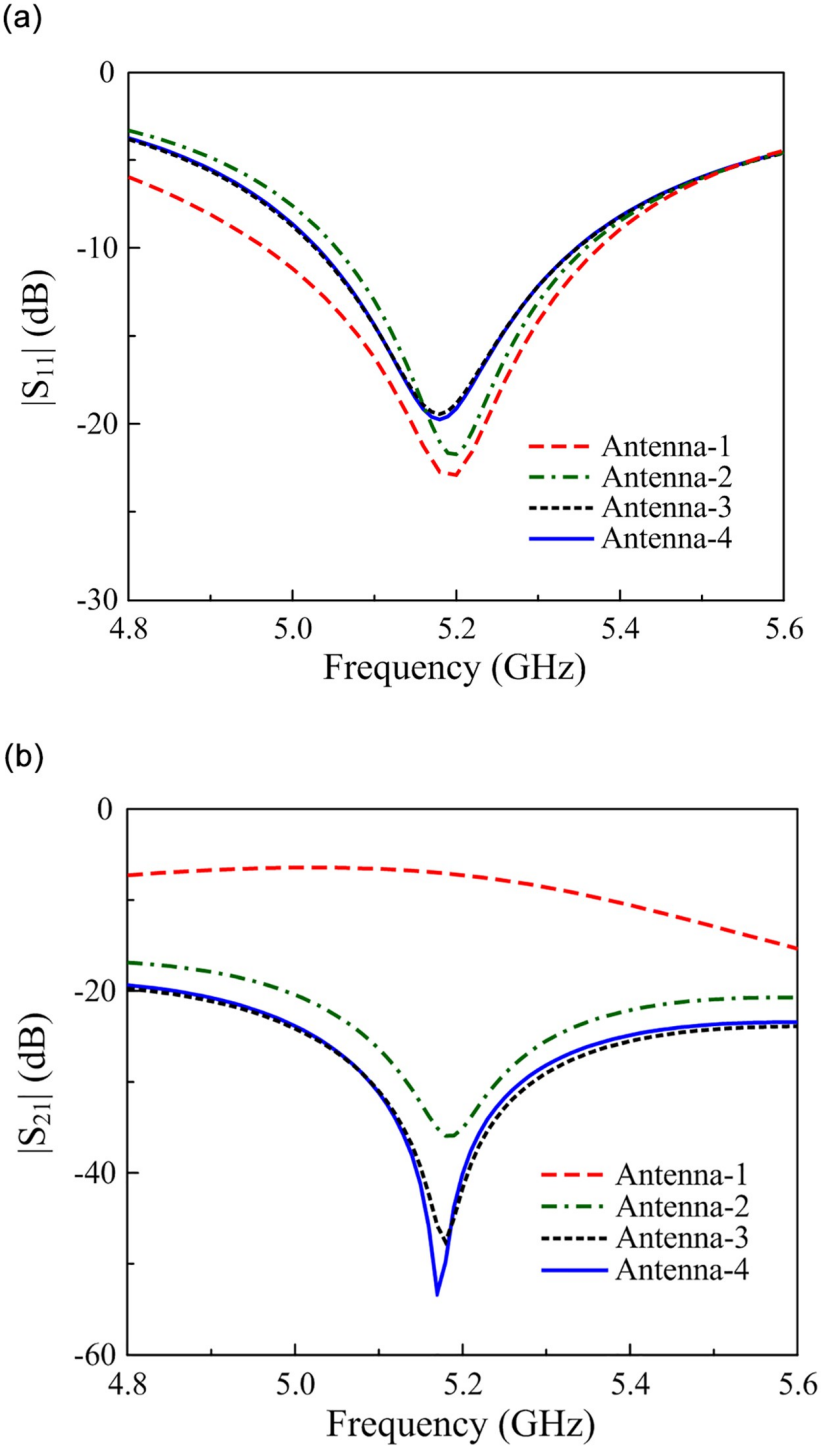
Simulated (a) |*S*_11_| and (b) |*S*_21_| of different antennas.

The simulated results in terms of the reflection coefficient (|*S*_11_|) and transmission coefficient (|*S*_21_|) of these antennas are illustrated in Fig 3. It can be seen obviously that the operating bandwidths of these designs are almost similar. Meanwhile, the antennas with a decoupling network show significant enhancement in the isolation aspect. Here, the isolation for Antenna-1 is around 8 dB. When one grounded stub is added, the isolation of Antenna-2 increases up to 36 dB. Further enhancement can be attained with more grounded stubs and the figure for Antenna-3 is about 48 dB and then the best value of 54 dB is obtained by Antenna-4.

**Fig 4:**
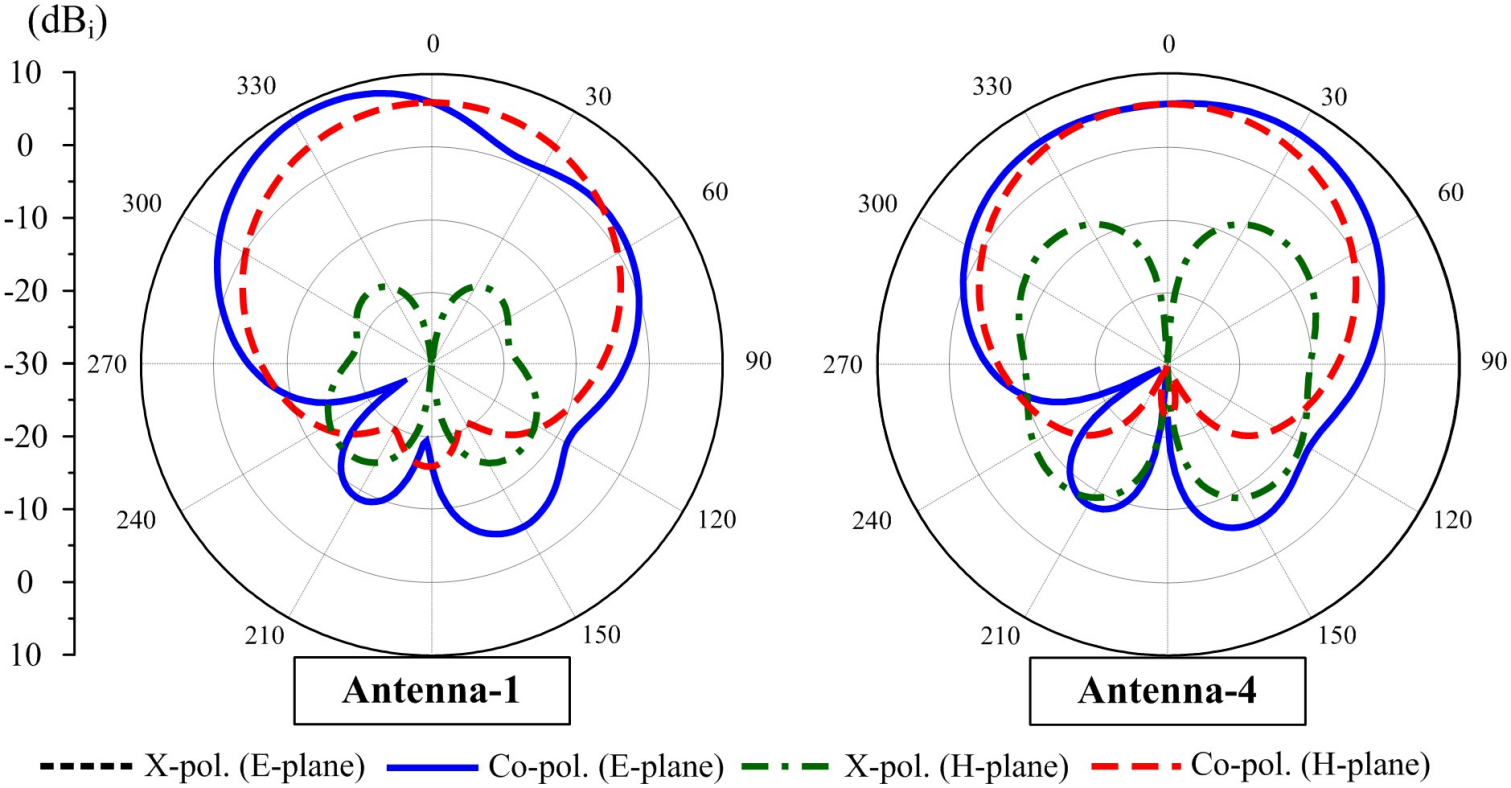
Simulated radiation patterns at 5.2 GHz.

In terms of far-field radiation patterns, Fig 4 shows a comparison between Antenna-1 and Antenna-4 at 5.2 GHz. For both cases, the radiation patterns in the H-plane are symmetric around the broadside direction (+ *z*). In the E-plane, the effect of the decoupling network is more significant. As observed, the pattern is considerably distorted on one side and deviated from the + *z* direction about 30° for the antenna without decoupling network. This is due to the unwanted radiation from the non-excited patch. On the other hand, the pattern of the decoupled antenna is almost symmetric around the +z direction. In this case, the induced current flowing on the non-excited element is very small, which minimizes the radiation from this element. However, the presence of the decoupling structure results in the current flowing along the stub, which increases the cross-polarization component of Antenna-4. Regarding the front-to-back ratio (FBR), it can be seen that the decoupled antenna has a higher FBR ratio. In other words, Antenna-4 has smaller back radiation than Antenna-1. It could be attributed that for Antenna-1, the high mutual coupling leads to strong radiation from the non-excited element. Thus, there are more diffracted waves occurring at the edges of the ground. In contrast, the diffracted waves of Antenna-4 are only generated by the excited element, leading to smaller back radiation.

### Antenna characteristics

**Fig 5:**
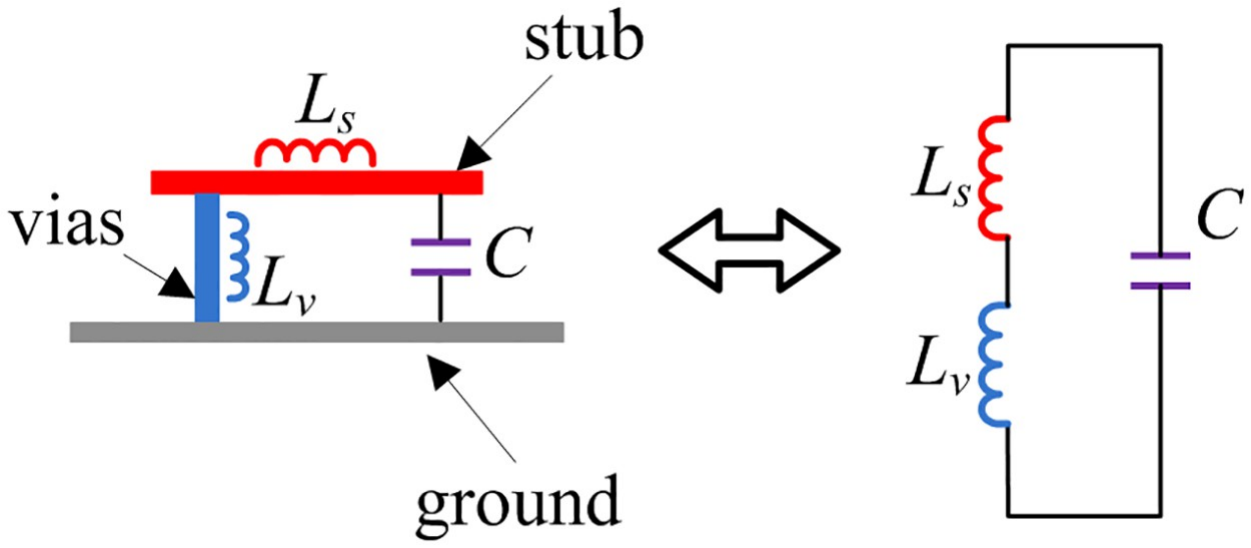
Equivalent circuit of the stub with shorting vias.

Based on the aforementioned studies, the function of the grounded stub as the decoupling network has been revealed. Fig 5 shows the equivalent circuit of the decoupling network. With the presence of the vias, the parallel LC resonator is created. Here, C denotes the capacitance between the stub and the ground. *L*_*s*_ and *L*_*v*_ are respectively the inductance of the stub and the shorting vias. The stub is chosen about a quarter-wavelength at the desired frequency. The quarter-wavelength shorted stub acts as a parallel LC resonant circuit with very high impedance. This LC circuit can provide a rejected frequency band and then, the coupling fields are blocked by this circuit [27, 28]. For Antenna-2, a single stub works as a single band-stop filter. For Antenna-3 and -4, two stubs are employed, and they are considered as two band-stop filters. However, these stubs are separated in Antenna-3 and connected in Antenna-4. Thus, it can be concluded that the decoupling structure utilized in Antenna-4 is more effective in coupling suppression than the structure employed in Antenna-2.

**Fig 6:**
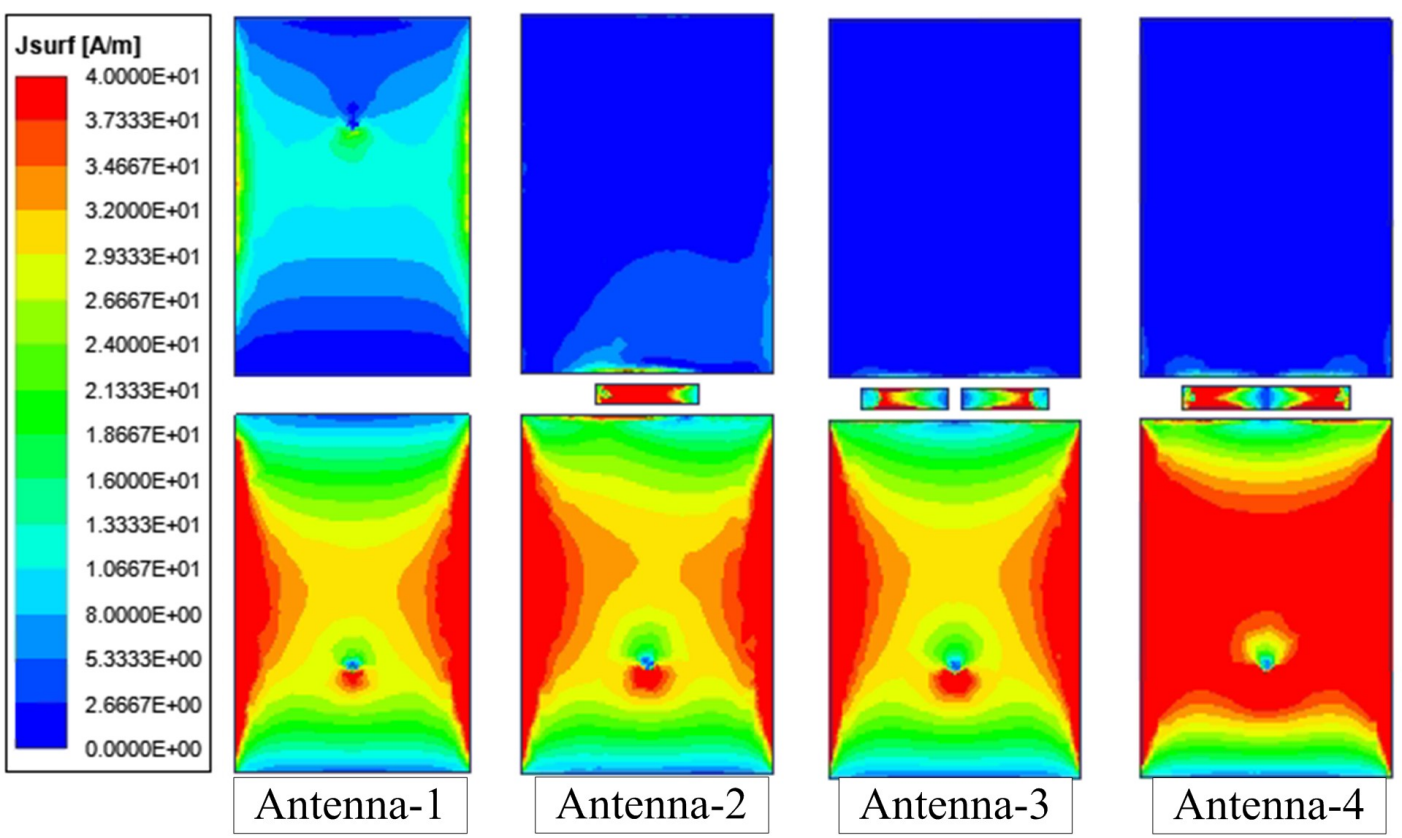
Simulated current distributions on different antennas at 5.2 GHz.

The coupling suppression can be demonstrated obviously by investigating the current distribution of the antennas with and without the decoupling network. Fig 6 shows the current distributions at 5.2 GHz of Antenna-1 to -4. As seen, there is a strong induced current flowing on the non-excited patch for the coupled antenna. In contrast, with the presence of the grounded stub, this induced current is significantly suppressed. In these cases, the current is strongly distributed on the decoupling networks. Note that the currents on the stubs of Antenna-2 and -3 are extremely low at the open side. This phenomenon is also observed for Antenna-4. This explains that the decoupling network of this design is equivalent to two band-stop filters.

**Fig 7:**
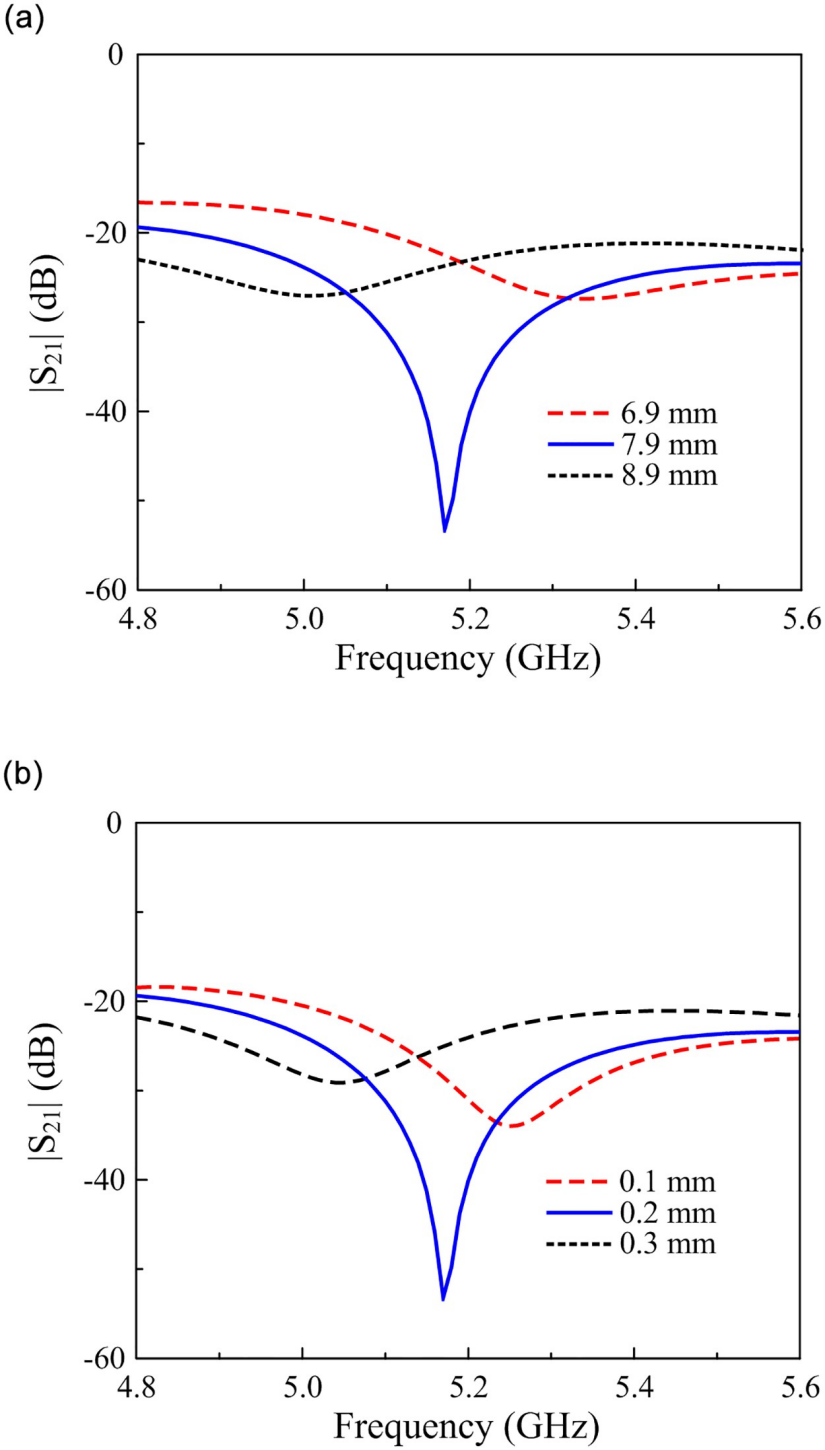
Simulated |*S*_21_| of the proposed two-port patch antenna (Antenna-4) against the variations of (a) *l*_0_ and (b) *r*_*v*_.

To design the decoupling network, the stub’s length, and the vias’ diameter determine the inductance and capacitance of the circuit. Thus, they have a significant effect on the stop band. Fig 7 shows the |*S*_21_| values of the proposed design against the variation of the stub’s length, *l*_0_, and the vias’ diameter, *r*_*v*_. Note that the matching performance is almost stable with the variations of *l*_0_ and *r*_*v*_ and thus, they are not shown to save space. As seen in Fig 7, these parameters have a strong effect on the coupling performance. The grounded stub acts as an *LC* resonant circuit, whose resonance is inversely proportional to the inductance (*L*) and the capacitance (*C*) values. For the proposed grounded stub, changing the size of the vias and the stub directly affect the inductance and the capacitance values. Thus, the resonant frequency is controlled, and the surface wave is blocked by this circuit. This is obviously illustrated through the low |*S*_21_| values.

## Measured results

**Fig 8:**
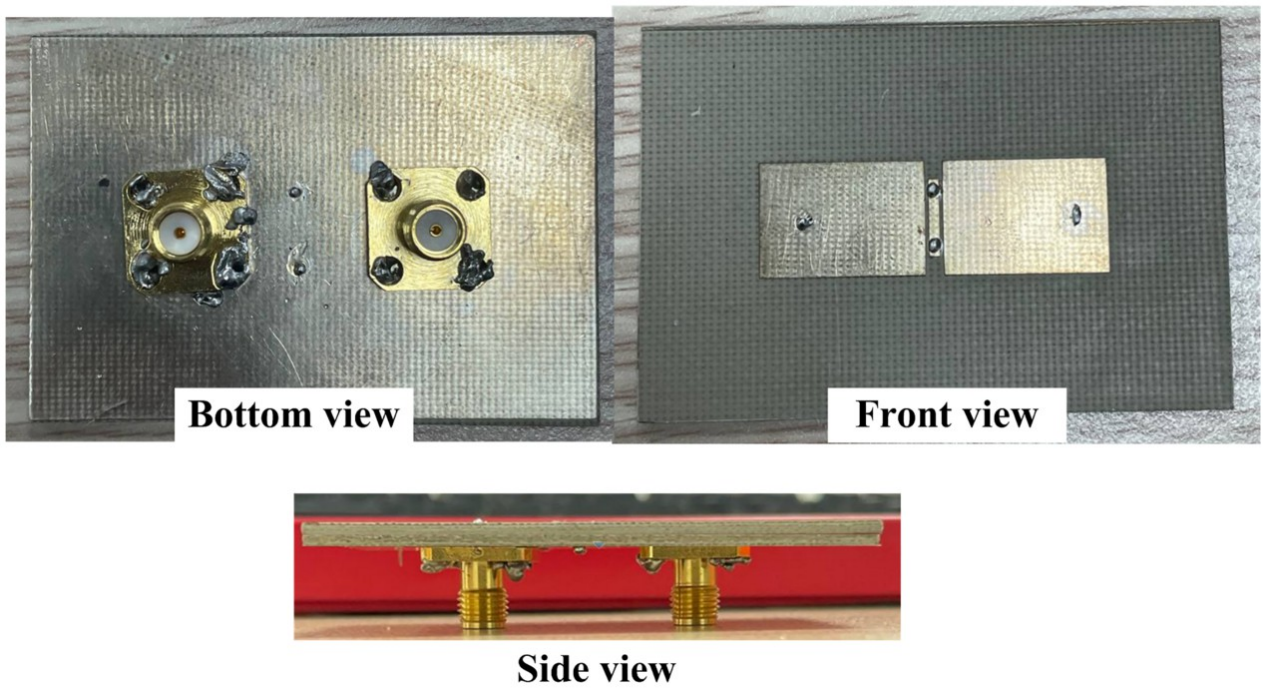
Photographs of fabricated antenna.

The measurement is implemented on a fabricated antenna prototype shown in Fig 8 to validate the design theory. An Agilent E8362B network analyzer is used to measure the S-parameters in an open-air condition. Far-field radiation measurements are carried out in an isolated anechoic chamber located at the Electromagnetic Wave Technology Institute in Seoul, Korea. The proposed antenna is used as a receiving antenna, while the standard wideband horn antenna is used as a transmitting antenna. Generally, the simulations and measurements are well-matched. The small difference could be attributed to the tolerances in fabrication and imperfection in the measured setup.

### S-parameter and far-field results

**Fig 9:**
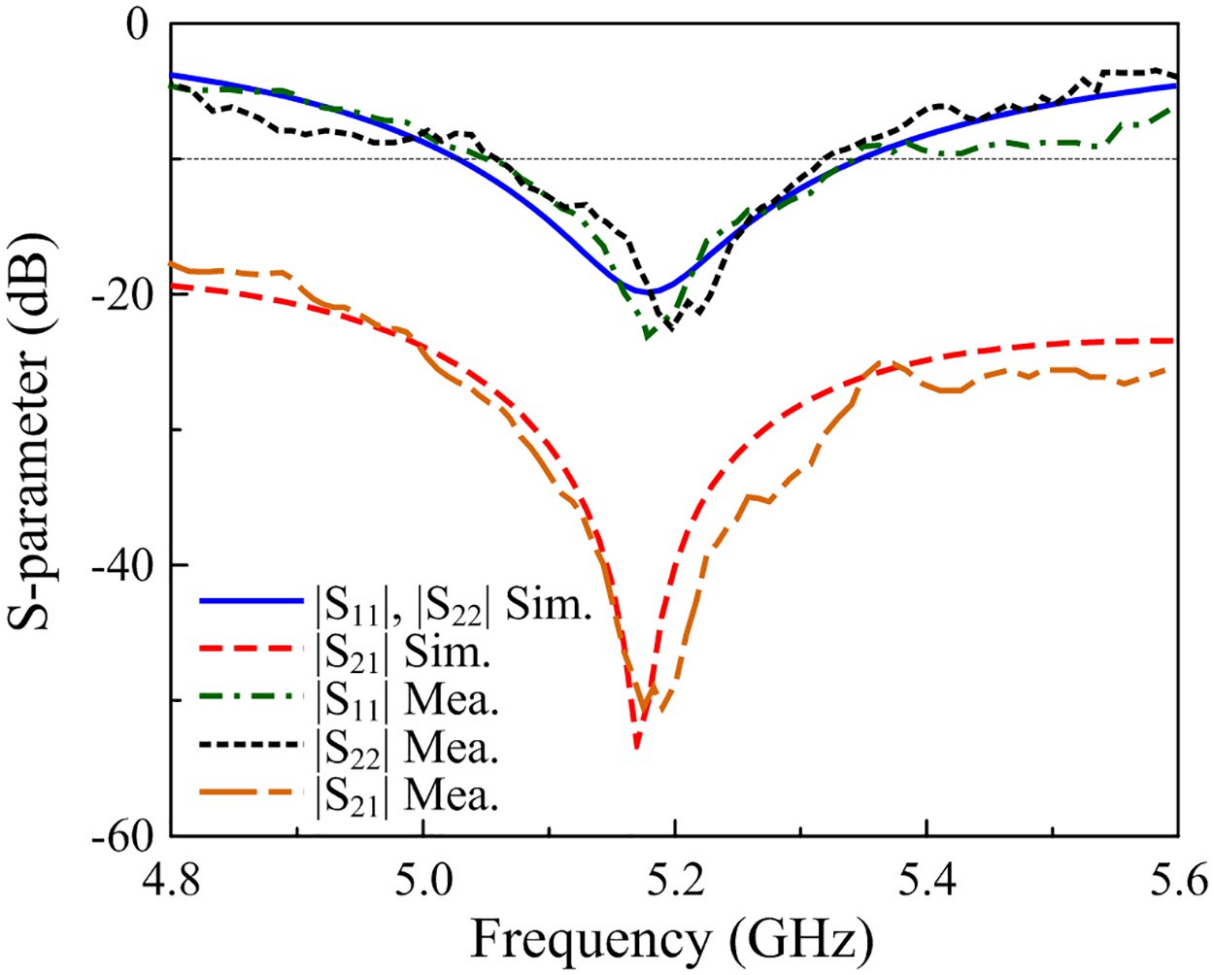
Simulated and measured realized gain of the proposed two-port patch antenna.

Fig 9 shows the simulated and measured S-parameter of the proposed two-port design. The presented antenna satisfies the requirements for impedance matching and port isolation in the frequency range from 5.06 to 5.32 GHz (5%), in which the measured |*S*_11_| is less than –10 dB and |*S*_21_| is smaller than –20 dB.

**Fig 10:**
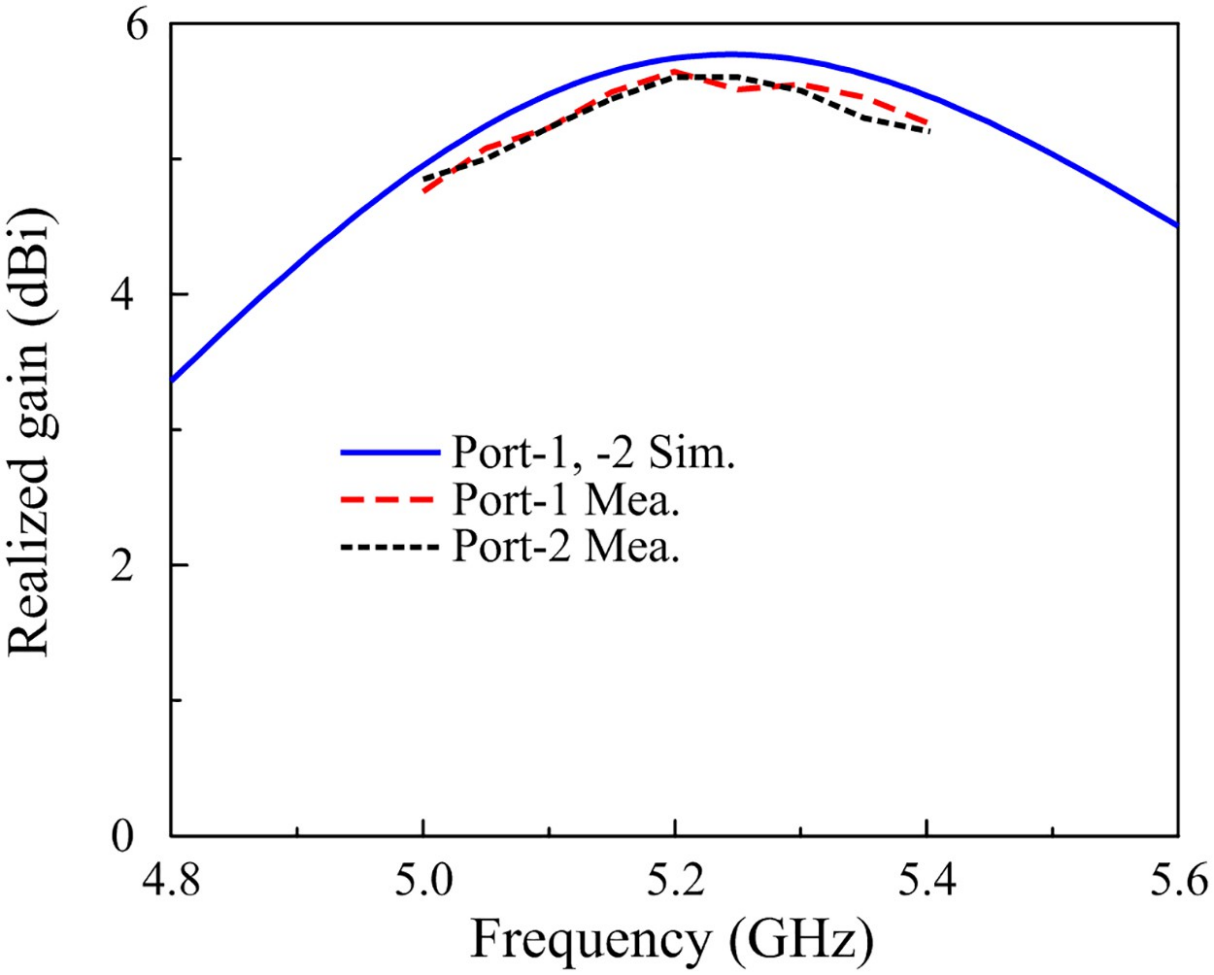
Simulated and measured gain radiation patterns at 5.2 GHz with Port-1 excitation.

Fig 10 presents the simulated and measured realized gain results in the broadside direction (+ *z*-direction). For the far-field test, when one port is excited, the other is terminated with a 50-Ω load. As observed, the measured gain values for Port-1 and Port-2 excitations are almost similar. The antenna has a maximum gain of 5.6 dBi and a minimum gain of 5.0 dBi within the operating band from 5.06 to 5.32 GHz.

**Fig 11:**
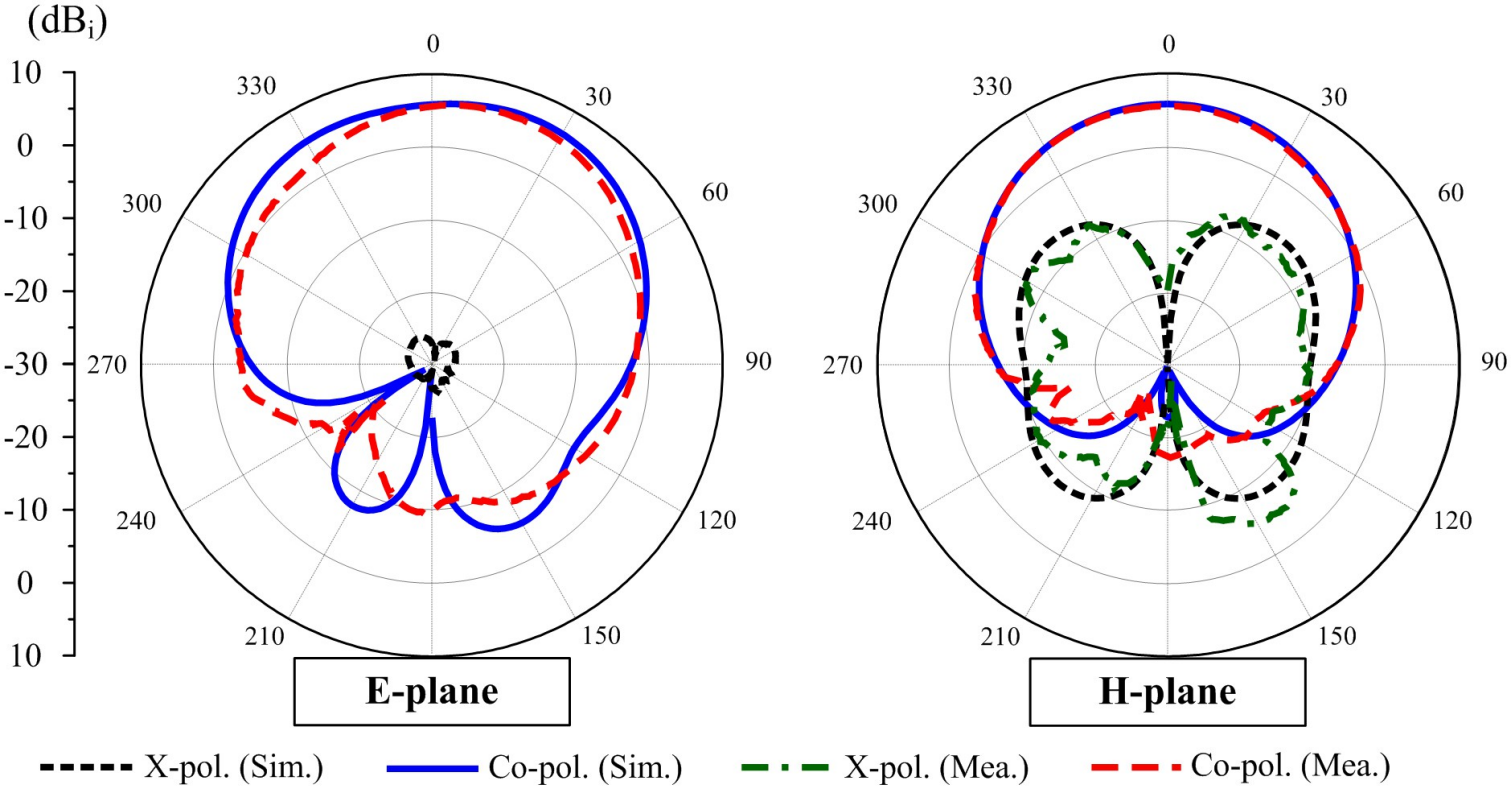
Simulated and measured gain radiation patterns at 5.2 GHz with Port-1 excitation.

The radiation patterns at 5.2 GHz of the proposed antenna with Port-1 excitation are plotted in Fig 11. At this frequency, the antenna achieves the best isolation. Due to the symmetric geometry, the patterns for Port-2 excitation are not presented to save space. It can be seen that in both E- and H-plane, the proposed antenna performs the boresight radiation.

### MIMO parameter

The envelope correlation coefficient (ECC) and the diversity gain (DG) are the most important MIMO parameters. They are calculated based on the S-parameter as Eqs (2), 3 and (4) [29].

$$\begin{array}{c} ECC_{ij}=\frac{{\left|R_{ii}^{*}*T_{ij}+T_{ji}^{*}*S_{jj}\right|}^{2}}{\left(1-{\left|R_{ii}\right|}^{2}-{\left|T_{ji}\right|}^{2})(1-{\left|R_{jj}\right|}^{2}-{\left|T_{ij}\right|}^{2}\right)} \end{array}$$ (2)

$$\begin{array}{c} ECC_{ij}=\frac{{\left|{\;\int \int \;}_{0}^{4\pi }[\vec{R_{i}}\left(\theta ,\varphi \right)\times \vec{R_{j}}\left(\theta ,\varphi \right)]d\Omega \right|}^{2}}{{\;\int \int \;}_{0}^{4\pi }{\left|\vec{R_{i}}\left(\theta ,\varphi \right)\right|}^{2}d\Omega {\;\int \int \;}_{0}^{4\pi }{\left|\vec{R_{j}}\left(\theta ,\varphi \right)\right|}^{2}d\Omega } \end{array}$$ (3)

$$\begin{array}{c} D_{gain}=10\sqrt{1-{\left|ECC_{ij}\right|}^{2}} \end{array}$$ (4)

**Fig 12:**
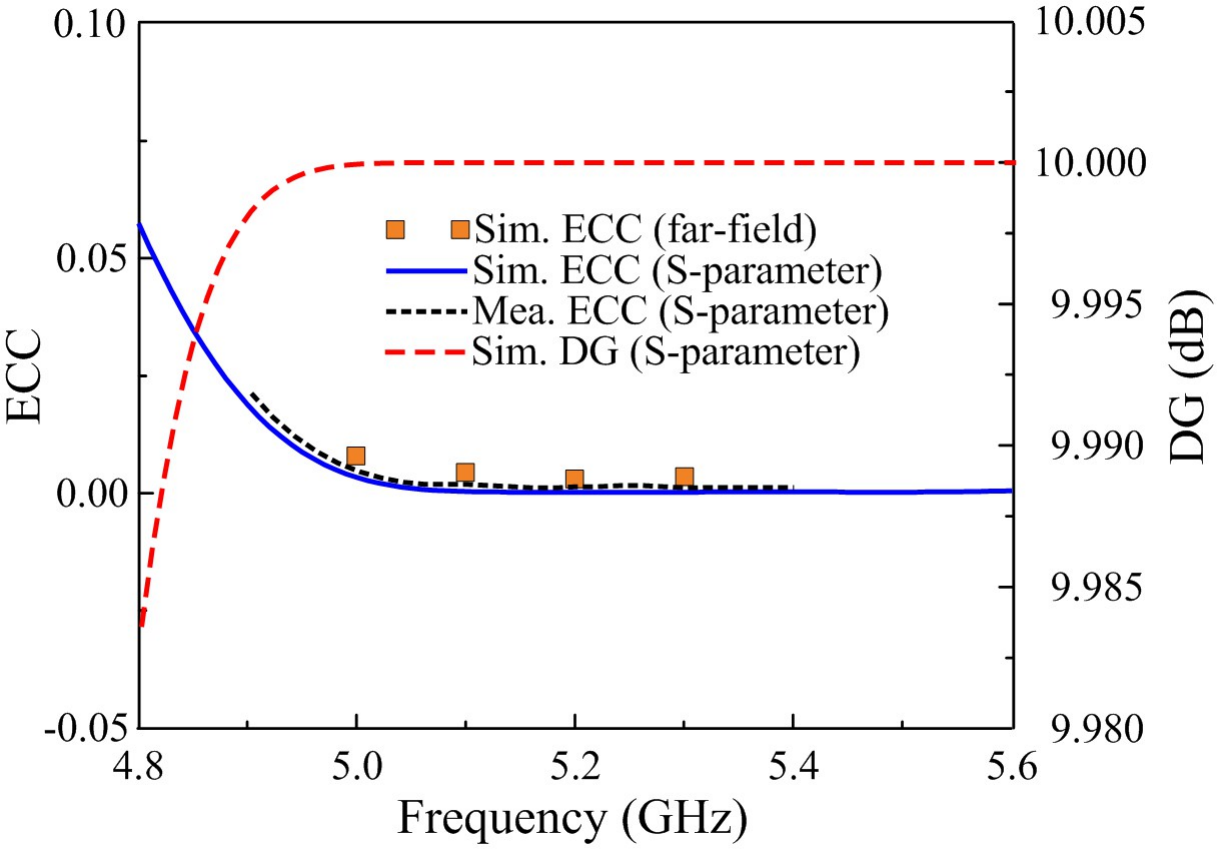
Calculated ECC and DG of the proposed two-port patch antenna.

Here, *i* and *j* are port numbers. *R* and *T* are the reflection and transmission coefficients. Ω is the solid angle of the far-field radiation patterns $\vec{R_{i}}(\theta ,\varphi )$ and $\vec{R_{j}}(\theta ,\varphi )$. The calculated ECC and DG results are illustrated in Fig 12. It can be seen that the DG is approximately equal to its theoretical maximum value of around 10. Besides, the ECC is much smaller than the acceptable value of 0.5 within the operating band.

**Fig 13:**
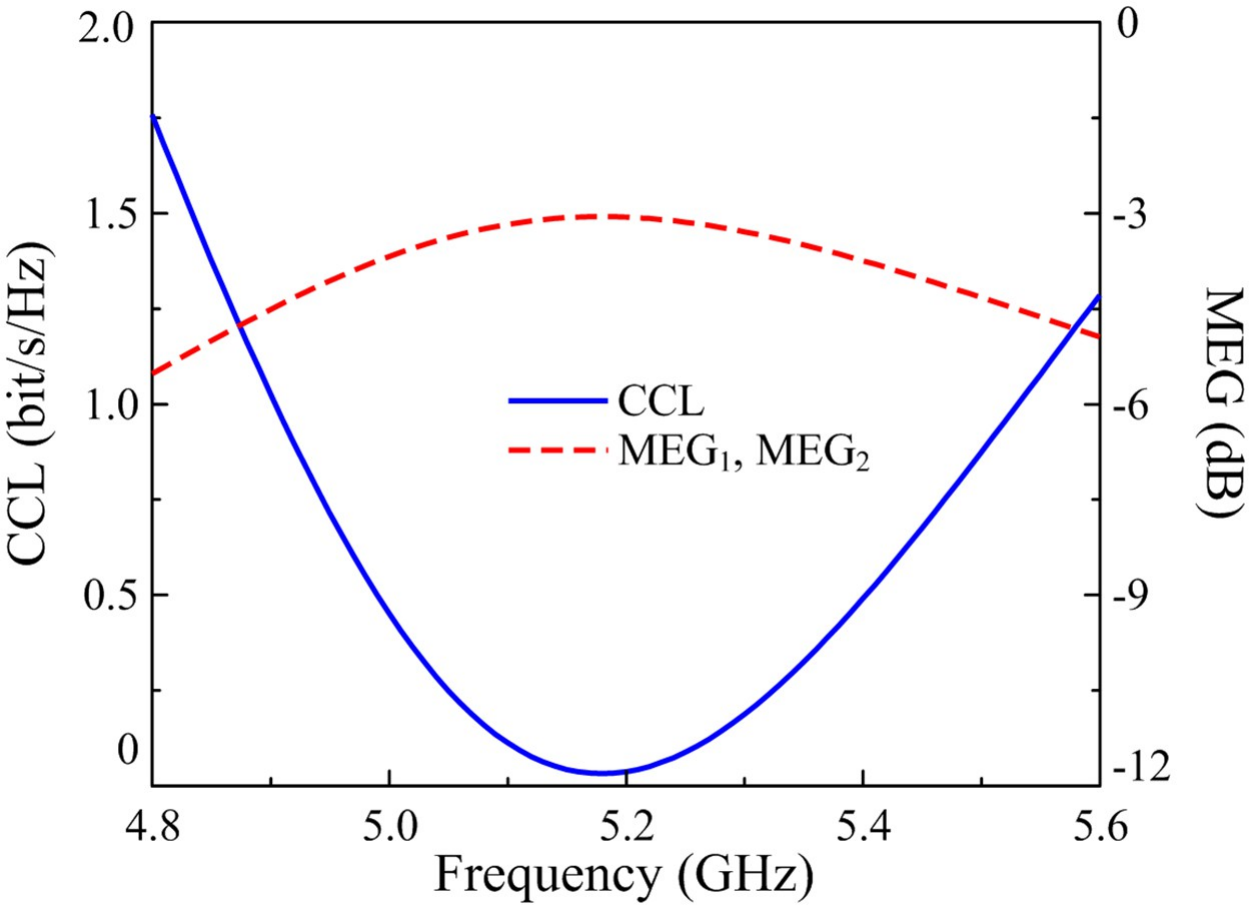
Calculated CCL and MEG of the proposed two-port patch antenna.

Further evaluations of MIMO performance are based on the CCL and MEG ratio parameters, as calculated in Eqs (5) and (6):

$$\begin{array}{c} \text{CCL}=-{\text{log}}_{2}\;\text{det}\;(\vartheta^{\mu }) \end{array}$$ (5)

where

$$\begin{array}{c} \vartheta^{\mu }=[\begin{array}{cc} \xi_{11} & \xi_{12}\\ \xi_{21} & \xi_{22} \end{array}] \end{array}$$

and

$$\begin{array}{c} \begin{array}{c} \xi_{11}=1-[{\left|S_{11}\right|}^{2}+{\left|S_{12}\right|}^{2}]\\ \xi_{12}=-[S_{11}^{*}S_{12}+S_{21}^{*}S_{12}]\\ \xi_{21}=-[S_{22}^{*}S_{21}+S_{12}^{*}S_{21}]\\ \xi_{22}=1-[{\left|S_{22}\right|}^{2}+{\left|S_{21}\right|}^{2}] \end{array} \end{array}$$

$$\begin{array}{c} MEG_{i}=0.5(1-\sum_{J=1}^{M}|S_{ij}|) \end{array}$$ (6)

where M is the number of antennas. As exhibited in Fig 13, the CCLs remain lower than 0.4 bits/s/Hz, and the figures for the MEG are similar for both ports. Those results claim that the proposed antenna has excellent characteristics to be applied in MIMO systems.

### Performance comparison

To demonstrate the effectiveness of the proposed method, Table 2 shows a comparison among the two-port antennas. It is noted that all the cited papers are the best in the literature. Although small element spacing can be achieved in [6, 8, 12], the use of NFR, MS, as well as strip wall significantly increases the antenna profile. The electromagnetic band gap (EBG) in [17] requires huge element spacing. Meanwhile, the methods proposed in [14, 21, 22] can achieve small spacing and low-profile configuration as well. However, the isolation improvement of such designs is lower than that of the proposed work. Additionally, the designs in [14, 21] employ the DGS technique, which has a drawback of high back radiation. Using the inductor [22] can achieve very small spacing, but low isolation enhancement. The proposed decoupling network shows the best isolation improvement with reasonable spacing between the elements. Furthermore, wider operating BW is another advantage of the presented antenna compared to the related antennas.

**Table 2:** Performance comparison among two-port antennas.

Ref.	Method	*ε* *	Overall size (λ_*c*_)	Spacing (λ_*c*_)	20-dB Iso. BW (%)	Max. Iso. (dB)	Iso. Impr. (dB)
[6]	NFR	2.6	0.75 × 0.60 × 0.22	0.035	1.5	35	28.4
[8]	MS	4.4	1.30 × 0.87 × 0.15	0.008	7.7	40	32.5
[12]	Strip Wall	3.5	0.86 × 0.58 × 0.22	0.03	1	54.3	45.2
[14]	DGS	3.5	1.16 × 0.83 × 0.03	0.03	1	54	34.3
[15]	DGS + stub	2.2	1.09 × 0.51 × 0.05	0.068	3.4	90	78
[17]	EBG	2.6	0.84 × 0.51 × 0.02	0.22	2.5	36	16
[21]	NL + DGS	4.4	0.84 × 0.51 × 0.02	0.018	4.6	50	40
[22]	Inductor	4.4	0.82 × 0.82 × 0.04	0.016	N/A	15.4	10.4
Prop.	Grounded stub	2.2	0.70 × 0.53 × 0.03	0.034	5	50	43

*: dielectric permittivity of the used substrates

## Conclusion

This paper investigates and presents a method to significantly suppress the mutual coupling of the closely packed two-port antenna. The design concept has been characterized in simulation and then validated by measurement. The measured results demonstrate that by using the grounded stub, the isolation of the two-element antenna is dramatically increased up to 50 dB. Compared to the antenna without the grounded stub, the isolation enhancement is about 43 dB. This improvement is much higher than the other related works in the literature. In addition, the proposed antenna also has high radiation efficiency of about 90% and good diversity performances in terms of ECC, DG, CCL, as well as MEG, are also attained by the proposed design.
